# Personalized public health: An implementation research agenda for the HIV response and beyond

**DOI:** 10.1371/journal.pmed.1003020

**Published:** 2019-12-31

**Authors:** Elvin H. Geng, Charles B. Holmes, Mosa Moshabela, Izukanji Sikazwe, Maya L. Petersen

**Affiliations:** 1 Division of Infectious Diseases, Department of Medicine and Center for Dissemination and Implementation, Institute for Public Health, Washington University in St. Louis, St. Louis, Missouri, United States of America; 2 Center for Global Health and Quality, Georgetown University Department of Medicine, Washington, DC; 3 Johns Hopkins School of Medicine, Baltimore, Maryland, United States of America; 4 School of Nursing and Public Health, University of KwaZulu Natal, Republic of South Africa; 5 Center for Infectious Diseases Research in Zambia, Lusaka, Zambia; 6 Division of Biostatistics, School of Public Health, University of California Berkeley, Berkeley, California, United States of America

## The third factor

We have arrived at a moment in the global HIV response where implementation research is ready to take center stage. Treatment has been scaled-up globally over the past ten years and, as a result, HIV-related mortality and incidence have fallen by more than 50% since a peak in the early 2000s [1]. Despite progress, however, antiretroviral therapy (ART) must still reach perhaps another 15 million persons living with HIV in the coming years. Progress in prevention also remains well below targets. The formula needed for success—past, present and future—consists of three main factors: first, the efficacy of available interventions to treat and prevent HIV; second, adequacy of financing; and third, the effectiveness, efficiency and quality of implementation. As to the first factor, well-tolerated, potent and affordable medications for HIV treatment and prevention already exist: tomorrow’s novel products will likely offer only incremental benefits, and do so through affecting barriers to implementation (e.g., injectable cabotegravir). Regarding the second, donor funding for the global HIV response has leveled out over the past decade and domestic allocations in low- and middle-income countries have not reached needed levels: a large surge in funding is unlikely [2]. Perhaps more than ever before, success in the global HIV response depends on the third factor in the equation: the effectiveness, efficiency, and quality of implementation.

## Heterogeneity in the HIV epidemic response

Progress in the global HIV response has brought new challenges on the path to full epidemic control into clearer view. Recent data show that, despite strong progress on average, the epidemic today is made up of a mosaic of microscopic epidemics and microscopic epidemic responses, which vary to a surprising degree in intensity and nature even within categories we assume are similar. For example, prevention efforts focus on high-risk populations such as adolescent girls and young women in Southern Africa, but these categories obscure large differences. Using latent class analysis, Nguyen and colleagues demonstrated that among adolescent girls and young women in South Africa only a minority (~20%) have sexual partners five or more years older, and this group had more than twice the rate of HIV acquisition as compared with others in the same sociodemographic categories [3]. Kaagayi and colleagues showed that within small geographic areas around Lake Victoria in East Africa, HIV prevalence varies from 9% to 43%—differences not explained by socioeconomic or community characteristics [4]. Cuadros and colleagues found that, even in a hyper-endemic community in South Africa, the majority of transmissions are linked to small hotspots [5]. By using a sampling-based approach to assess mortality (normally hidden by loss to follow-up) in Zambia, Holmes and colleagues found that 2-year mortality after starting ART varied from less than 5% to nearly 25% across 30 public facilities—again, differences that were not explained by obvious sociodemographic, clinical, health systems nor geographic factors [6]. Sikazwe and colleagues found that failures of retention varied by over 4-fold across the same facilities, with patients reporting a broad range of psychosocial (e.g., depression), structural (e.g., transport) and clinic-based (e.g., waiting times) barriers (alone and in combinations) [7]. In sum, across a range of patient, health systems, and community categories, a closer look reveals surprising differences.

The much anticipated “universal test and treat” studies published this past year may also have suggested heterogeneity of the effects of universal testing and treatment strategies on HIV prevention. In PopART [8], a cluster randomized trial done in Zambia and South Africa, Hayes and colleagues found that the study populations with the greatest population level viral suppression (Arm A) nevertheless experienced higher incidence than a study population with less suppression (Arm B). While chance differences in measurement, migration, or other artifactual reasons could explain this finding, another possibility is that this is a consequence of the different approaches taken to testing and treatment. Although the idea that reduced population-level viremia reduces HIV incidence is essentially incontrovertible and the UNAIDS 90-90-90 targets offer a universal benchmark for progress, these findings may also suggest that for any given level of suppression in a community, who is suppressed—which depends on local and community-specific factors in engagement, treatment access and retention—could greatly influence the effect of treatment as prevention.

## Adaptation in implementation

Today’s heterogeneity in the HIV epidemic and response illustrates a challenge at the heart of implementation research. Does the diversity of micro-epidemics and micro-responses imply that strategies to test, start and retain patients on treatment must be local? Does heterogeneity diminish the prospects of generalizable scientific insights in the implementation of the HIV response? No single approach, strategy or intervention will be needed by all (and will therefore be inefficient if universally delivered) nor useful for all (and will therefore fall short of helping all in need). Yet implementation research must deliver widely applicable insights to succeed in its aspirations. Heterogeneity poses both a crucial challenge for the scientific response to the HIV epidemic as well as implementation research more generally.

Novel scientific tools for uncovering and responding to heterogeneity are emerging in implementation research, and have implications for the HIV epidemic response. Approaches that tailor strategies to contexts fall under the umbrella of adaptation, a concept that may simultaneously address the priorities of meeting diverse needs and minimizing costs. While these aspirations are not new—PEPFAR’s field strategy is based on targeting resources to burden and mathematical models have provided proof of concept—a cohesive scientific approach to making and understanding adaptations would indeed be new [9]. One view is that implementation strategies should have both a conserved “core”, which would be designed to overcome as many of the vagaries of context as possible, as well as a malleable “periphery” that would be tailored to fit context. Chambers and colleagues have advanced the conceptualization of adaptation and see adaptation as the linchpin to sustainability: they decry the viewpoint that an intervention or strategy should stay the same in all settings to be effective, but rather see sustainability as the consequence of a continuous process of adaptive fit [10]. They suggest furthermore that the science of adaptation requires an “adaptome” or a systematic approach to capture and categorize adaptations as an empiric research agenda [11].

Experimental study designs that use sequential randomization can enable efficient empiric comparisons of particular adaptations [12], and therefore inform how to adapt interventions, with immediate relevance for public health innovations to address HIV. Sequential randomization allows investigators to ask, for example, which evidence-based interventions, used in what sequences, triggered by which events, lead to the best outcomes. Adaptive strategies that respond to patient characteristics, including preferences, reflect a recognition that the most relevant questions in the epidemic are not which biomedical intervention will work best, but instead which strategy of engaging populations in interventions over time will work best, and reframes priority research questions. In general, by pegging selection of interventions to response, adaptive interventions will be more effective (by escalating or changing interventions in those not responding) and more efficient (by maintaining an intervention when it is sufficient) as compared to a single intervention for all. Instead of assessing the effectiveness or comparative effectiveness of service innovations, for instance, an adaptive framing would ask whether, for example, an “induction–maintenance” strategy that starts with home visits and then de-escalates to a light touch model (e.g., adherence clubs) once initial adherence is established compares favorably to a “react and respond” model where patients start with minimal support but are offered intensified counseling if they do poorly (i.e., how to adapt to optimize effectiveness and efficiency). Or, instead of asking whether injectable pre-exposure prophylaxis (PrEP) is as effective as oral PrEP (a comparative effectiveness research question with no clear public health implications), an adaptive approach would allow researchers to assess “mosaic” effectiveness of injectable PrEP when introduced into an environment where oral PrEP is available by asking who should be offered oral or injectable PrEP, what criteria would be used to recommend switch to the other formulation, and what kinds of populations might respond distinctively to any particular sequencing strategy. By pegging changes in treatment to response, adaptive strategies react directly to the behavior of individuals, even when those behaviors are not prevalent in their sociodemographic groups. Modern statistical and machine learning approaches are also now available for efficiently and flexibly both detecting such heterogeneity and tailoring intervention delivery in response [13–15]. These tools can further be integrated into study designs to allow randomization probabilities themselves to evolve as heterogeneity in response is discovered during the study—in other words, to increasingly assign patients to the intervention likely to work best for them [16–21].

Techniques to fit interventions to particular contexts offer a different approach to the use of adaptation to overcome heterogeneity. These include choice experiments, widely used in marketing and based on rational utility theory, which are surveys in which a participant is offered two (or more) versions of a good or service where the attributes are varied. Repeatedly selecting the version they prefer [22] demonstrates an end-user’s otherwise unobserved preferences, the strength of those preferences, and the relative importance of those preferences against each other. Findings tell implementers how to shape delivery of evidence-based interventions (e.g., ART, PrEP) to be most acceptable, adoptable, and sustainable as used. For example, a choice experiment suggested that most HIV patients would most prefer infrequent encounters with health workers, but somewhat surprisingly do not mind the location of those visits if infrequent [23]. Llewellyn and colleagues showed that among MSM in London, 86% found face-to-face testing highly acceptable, but a subset of 14% preferred to test anonymously, at home and at a place and time of their convenience—suggesting an important potential role for self-testing [24]. Human- or user-centered design represents another set of techniques that can tailor interventions to particular organizational environments. User-centered design eschews the traditional research–participant dichotomy for the concept of co-creation, where end users and producers work iteratively to ideate, refine, and test how a particular product, service, practice, or technology fits with the values, priorities and preferences of end users [25]. These techniques can be invoked to provide local insights where differences are felt to be large enough to warrant more investment.

## A step toward personalized public health

Looking backwards, the scientific trajectory in the HIV response has been a remarkable microcosm of the entire translational scientific process. The field started with identifying the etiologic agent, understanding molecular mechanisms, then developed medications, tested and refined over decades of trials, and studied testing and adherence. During this process, the scientific community addressing HIV has had to pivot many times—to incorporate novel scientific approaches to answer new classes of questions. Looking forward towards the finish line, the scientific response must pivot once again. The hallmark of heterogeneity in the response today requires not only more science but different science. The future of public health must address not only the adaptation of systems and services, but also advance a scientific agenda to elevate the patient experience, and to incorporate patient-reported outcomes and novel approaches to patient activation. Together, these novel directions would seek to make the response more personalized and tailored to those being left behind. Getting to the finish line, at least in public health, is not about who gets there first, but what we can do to make sure that all arrive. Bringing personalized public health into practice will require us to turn the idea of meeting patients halfway from a notion into a genuine scientific agenda.
